# Proton Conducting Membranes with Molecular Self Assemblies and Ionic Channels for Efficient Proton Conduction

**DOI:** 10.3390/membranes12121174

**Published:** 2022-11-22

**Authors:** Avneesh Kumar, Dong Wook Chang

**Affiliations:** Department of Industrial Chemistry, Pukyong National University, Busan 48513, Republic of Korea

**Keywords:** supramolecular assemblies, molecular columns, molecular wires, ionic channels, proton conductors, fuel cells

## Abstract

Supramolecular assemblies are vital for biological systems. This phenomenon in artificial materials is directly related to their numerous properties and their performance. Here, a simple approach to supramolecular assemblies is employed to fabricate highly efficient proton conducting molecular wires for fuel cell applications. Small molecule-based molecular assembly leading to a discotic columnar architecture is achieved, simultaneously with proton conduction that can take place efficiently in the absence of water, which otherwise is very difficult to obtain in interconnected ionic channels. High boiling point proton facilitators are incorporated into these columns possessing central ionic channels, thereby increasing the conduction multifold. Larger and asymmetrical proton facilitators disintegrated the self-assembly, resulting in low proton conduction efficiency. The highest conductivity was found to be approaching 10^−2^ S/cm for the molecular wires in an anhydrous state, which is ascribed to the continuous network of hydrogen bonds in which protons can hop between with a lower energy barrier. The molecular wires with ionic channels presented here have potential as an alternative to proton conductors operating under anhydrous conditions at both low and high temperatures.

## 1. Introduction

Nature has taught us how noncovalent interactions, such as hydrogen bonds and ionic interactions, play a key role in controlling the order within the molecules via self-assembly, thus affecting the biological functions [1,2,3,4]. Biological systems have effective proton conducting arrays consisting of proteins where molecular communications are responsible for its proper action [5,6]. With regard to the artificial materials used in organic electronics for electron conduction, the columnar type of structure has been reported to be preferable for their (electronic) charge transporting properties [7,8].

Proton conductors, for instance, Nafion, have been reported to have interconnected ionic channels that are directly responsible for their higher proton conductivity as a result of the participation of carrier molecules [9,10]. However, in polymer-based conductors, the ionic channels may not be interconnected throughout the network as the self-assembly in polymers is complex globally, as well as locally, and cannot be accomplished precisely. Hence, the proton conduction in specific dimensions cannot be studied very well [11,12]. In addition, water molecules are also essential for such interconnected ionic channels in these polymers e.g., sulfonated polymers. Therefore, there remains a challenge with ionic polymers in the obtaining of high proton conduction in the absence of water and without dissociation of the ionic channels. In this regard, small molecules with comparatively less complex chemistry, with a proper fraction of ionic and nonionic groups, can be introduced, targeting a well-defined supramolecular assembly with a specific shape with more precision to meet the demands [13,14]. In supramolecular assemblies of small molecules, the risk of disruption of the ionic channels can be prevented by creating a proton-conducting molecular wire where the conduction is increased multifold and can be investigated with better understanding [15]. Proton facilitators have been used previously in combinations of polymers or other self-assembled systems to improve the proton conduction, as the boiling point of these facilitators is greater that of water [16,17]. However, further correlation between the molecular architecture and proton conduction either in one direction or another is still of greater interest and must be explored in order to develop thermally and mechanically stable proton conducting molecular wires that can operate above 100 °C for a longer period of time. 

**Scheme 1:**
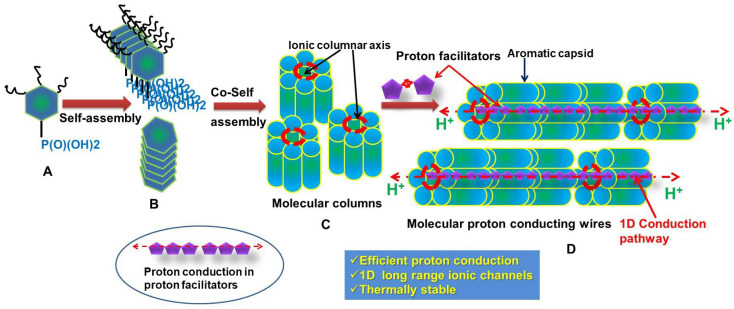
(**A**) A symmetrical organic phosphonic acid with long alkyl chains self organizes or undergoes supramolecular assembly in the solid state, producing multiple stacks of molecular units (**B**); that further co-assemble and result in columns of molecular units (**C**); in the next decisive step, the proton facilitator is added to these molecular columns where proton conduction takes place in one dimension within the interconnected ionic channels. Molecules of proton facilitator incorporate themselves into the already formed ionic channels via hydrogen bonds; this entire process results in the formation of long-range molecular proton-conducting wires (**D**) consisting of the bundles of the molecular columns and the molecules of proton facilitator.

Herein, we report an innovative and simplified approach to engineer long range and unidirectional ionic channels with increased proton conduction in the absence of water. These ionic channels are formed simultaneously upon the self-assembly and co-assembly of the proton facilitators, while the proton conduction is raised tremendously in a ‘cooperative’ manner. A schematic presentation of our current innovation and its impact is given in Scheme 1. As highlighted, a proton conducting organic phosphonic acid with negligible conductivity in the dry state was selected because it can form discotic columns, as described earlier in the literature [16]. In Scheme 1, the organic phosphonic acid A undergoes a self-assembly process leading to stacking of the molecules where the ionic or proton conducting groups (phosphonic acid) turn towards the axis of the columns. In the next step, the proton facilitators are added to these molecular columns for further co-assembly; the molecules of proton facilitators incorporate themselves into the axis of the columns and contribute towards the increase in proton conduction by forming a continuous hydrogen bonded network. Finally, the molecular wires with unidirectional ionic channels are formed where the outer shell or capsid is made up of aromatic rings and alkyl chains.

The obtained molecular wires are stable up to a higher temperature and conduct protons efficiently and ‘cooperatively’, without the requirement of water. Although the proton facilitators have their own network for proton conduction, they lack any well-defined architecture, thus they cooperate with the phosphonic acid groups of the parent molecules forming the discotic columns. This results in the formation of molecular wires with ionic channels and increased proton conductivity. The major attributes of these molecular wires with unidirectional and interconnected ionic channels are: (1) they conduct protons efficiently in one dimension (1D) without any disruption of their pathway, (2) the molecular columns in these wires are stable in a range of temperatures; thus, the proton conductivity can be associated directly with the columnar morphology and ionic channels, and (3) these molecular wires can be fabricated easily on an electrode surface without any disintegration of the supramolecular assembly. 

## 2. Result and Discussion

**Figure 1:**
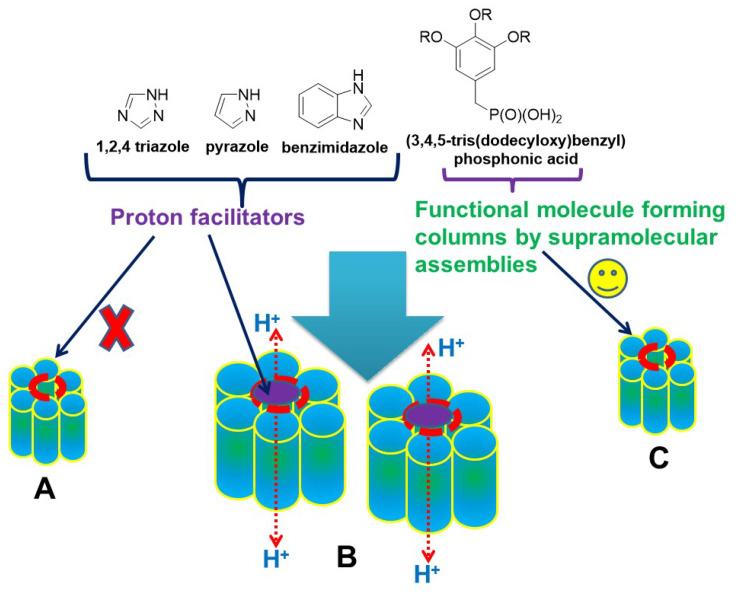
Chemical structure of three different proton facilitators with distinct acidity and organic phosphonic acid molecules (shown above). (**A**) Due to the absence of a distinct chemical phase, the proton facilitators are unable to produce any well-defined ordered shape; however, (**B**) these facilitators can integrate into the ionic channels and allow rapid proton conduction in one-dimension in (**C**) the molecular columns formed by the organic phosphonic acid **1**.

Proton facilitators such as pyrazole and benzimidazole conduct protons via a similar process, e.g., proton hopping occurring in the phosphonated proton conductors (Figure 1) [18,19]. However, proton relocation in the case of triazole occurs via a special process called tautomerism [20]. The fast proton mobility is usually associated with the distance between the hopping sites and the activation energy. Due to their high boiling point, these facilitators can replace water which is known to contribute towards the higher proton conduction in sulfonated membranes in a certain temperature range.

However, these facilitators lack any well-defined, ordered nanophase separation, therefore a deep understanding of their proton conduction in supramolecular assemblies and ionic channels cannot be investigated. In contrast, the organic phosphonic acid **1** with long alkyl chains, a rigid core and an ionic group has the ability to undergo phase separation, evolving as the molecular columns form via noncovalent interactions such as hydrogen bonds and pi–pi bonds (Figure 1). When organic phosphonic acid **1** and proton facilitators are combined together in the solid state, the properties and phenomena related to the two different molecules add together in a ‘cooperative’ manner in which the molecular columns and the higher proton conduction are achieved in a holistic way. 

The rapid proton conduction is achieved by the proton facilitator, whereas the molecular columns with ionic channels are born from the organic phosphonic acid (Figure 1). The formation of the acid-base complex in these assemblies was investigated by FT-IR spectrometry. In the FT-IR spectrum (Figure S1), the appearance of an absorption band at 1150 cm^−1^ was attributed to the phosphonyl group (P=O) of the organophosphonic acid which disappeared in the spectrum upon mixing of the acid and the proton facilitator. Additionally, the absorption bands at 939 and 1030 cm^−1^, corresponding to a symmetric and an asymmetric vibration of P-O in the organophosphonic acid, also decreased. Furthermore, the absorption band at ca. 3120 cm^−1^ which was associated with the stretching band of N-H group decreased relative to the pure pyrazole unit. These observations indicate that the P-OH group interacts with the pyrazole unit through hydrogen bond formation. Obviously, a rather broad peak at 1700–2500 cm^−1^ for the acid-base complex was also noted, due to the non-covalent interactions between the nitrogen and oxygen atom of pyrazole and organic phosphonic acid, respectively [S1]. 

As described earlier, the proton conductivity in these molecular wires predominantly depends upon the extended hydrogen bond network between the proton facilitators and molecular columns formed during the process. Therefore, it is necessary to study any change or modification in proton conduction mechanism as it can provide us with an insight into proton sites and the presence of a charge carrier, if any.

**Figure 2:**
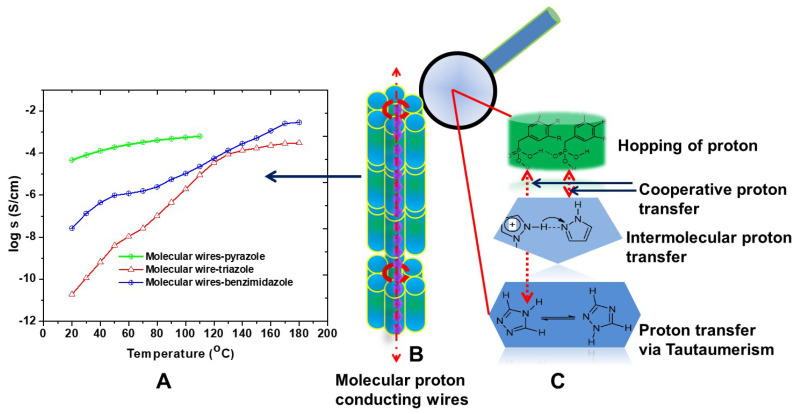
(**A**) Proton conductivity of molecular wires in the absolute dry state, (**B**) individual molecular wires and (**C**) proton transfer processes in proton facilitators and cooperative proton hopping between the proton facilitators and organic phosphonic acid **1**.

**Figure 3:**
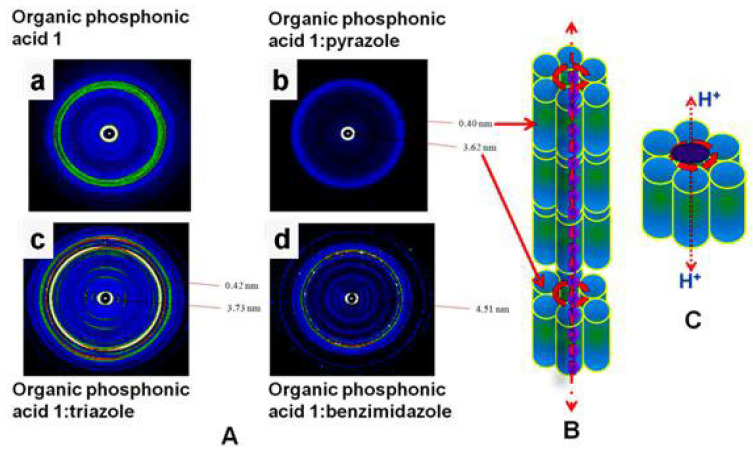
(**A**) 2DWAXS diffraction patterns recorded for (**a**) organic phosphonic acid **1**, (**b**) organic phosphonic acid **1** and pyrazole, (**c**) **1** and triazole and (**d**) **1** and benzimidazole at 30 °C; (**B**) illustration of molecular wires and (**C**) an individual molecular column.

In Figure 2, the proton conductivity in these molecular wires with proton facilitators is given, along with a depiction of the proton transfer processes taking place among the facilitator molecules and organic phosphonic acid **1**. For the blend of the organic phosphonic acid **1** and pyrazole, an increased value for the conductivity, approaching up to 4.8 × 10^−2^ S/cm, was observed above 90 °C (Figure 2A). In addition, the conductivity was found to be temperature dependent. It has been reported that diazoles (pyrazole, imidazole) can be incorporated in the interlayer region of the phosphonic acid group as a monolayer, and thus can increase the conductivity [21,22,23]. The replacement of pyrazole and imidazole with the corresponding mono- and di-methyl or ethyl substituted derivatives lowered the conductivity by at least one order of magnitude with a corresponding increase in activation energy. Unexpectedly, any sharp rise in the conductivity of the blend of organic phosphonic acid **1** with triazole and benzimidazole was not noticed. Similarly, the lower conductivity of molecular wires composed of organic phosphonic acid **1** and triazole and benzimidazole at 30 °C was associated with the lack of a monolayer of the guest molecules (triazole, benzimidazole) and the higher resultant activation energy (Table 1, Entry 3, 4). On the contrary, the conductivity began to rise sharply with temperature (4.8 × 10^−2^ S/cm at 90 °C) for the molecular wires of organophosphonic acid **1** and pyrazole. The possible reason for this could be that pyrazole molecules in the dynamic phase generated by heat energy can interact strongly with the phosphonic acid groups and thus enhance the proton conductivity by lowering the proximity between two phosphonic acid groups. Such proximity of the nearby protons of phosphonic acid groups is also minimized by the orientation of pyrazole molecules in ionic channels. It is assumed that due to the planarity and symmetry of pyrazole molecules, their position in ionic channels was thermodynamically favorable for exchanging protons with phosphonic acid groups. Additionally, the orientation of pyrazole molecules within the ionic channels may have also prevented phosphonic acid from suffering proton loss due to the formation of P-O-P bonds (anhydride) at high temperatures, as has been reported for phosphonated proton conductors [24,25,26]. In triazole molecules, the proton dynamics due to tautomerism can be linked to the lower proton conductivity, as the minimum number of protons is available for interacting with the phosphonic acids, and therefore not all triazole protons may be participating in the ionic channels. Some of the protons may have engaged with the clusters of the triazole molecules [27]. For molecular wires with benzimidazole, although the benzimidazole seems to have a benzene ring for pi–pi interactions, due to its unsymmetrical shape and geometry it may have acted in a rather different manner; some of its molecules may have remained free from the phosphonic acid groups and may not have cooperated in the co-assembly leading to the molecular columns. This is also shown by the 2DWAXS of benzimidazole in which no crystalline order phase was observed when compared to the organic phosphonic acid **1** (Figure S2). For the 2DWAXS of molecular wires with benzimidazole, a similar pattern to the isolated benzimidazole molecules was recorded, clearly indicating that the free benzimidazole molecules may have intercalated in the alkyl chains, thus having no impact on conductivity (Figure 3d and Figure S2). From the above findings, it can be said that the molecular wires with pyrazole have shown the highest intrinsic anhydrous conductivity, whereas the wires with benzimidazole appear to be less efficient, with the conductivity remaining at 10^−8^–10^−6^ S/cm up to 120 °C. Furthermore, the dissociation constant (*p*Ka) value of the corresponding organic proton facilitator can also affect the proton transfer and thus influence proton conduction [28]. As shown in Table S1 (SI), the 1H-1, 2, 4-triazole (*p*Ka = 2.2) is much more acidic than pyrazole (*p*Ka = 2.5) and benzimidazole (*p*Ka = 8.52), which further accounts for the high proton conductivity of the organophosphonic acid **1**-pyrazole in the temperature range of 30–90 °C (Figure 2A). The proton conductivity of different molecular wires can also be associated with the number of protonation sites in proton facilitator molecules. In fact, a molecule with two protonation sites has been reported to assemble into molecular clusters consisting of ~20 molecules linked via intermolecular hydrogen bonding [28]. Therefore, the molecular wires of the organophosphonic acid **1** with pyrazole have shown high conductivity at 180 °C due to the fast proton transfer process. Bio-inspired anhydrous proton conductors such as self-assembled acid-base complexes of mono-dodecylphosphate (MDP) and 2-undecylimidazole (UI) with long hydrophobic chains have been reported in the literature [29]. These acid-base composites exhibited the lamellar type of self-organization with an effective proton conduction pathway. The proton conduction in these composites was driven by the Grotthuss mechanism [30]. In this mechanism, the proton translocation occurs from one site to the other in a ‘wire’ consisting of a dynamic hydrogen bonded network. Proton diffusion is accompanied by structural changes in the molecules [31,32]. The activation energies (***Ea***) for these composites were reported to be 0.22–0.34 eV. The activation energies (***Ea***) for these molecular wires were estimated by the slope of Arrhenius plots from the proton conductivity data and by employing the Arrhenius equation as follows:

(1) $$\sigma =\sigma 0\exp\;(-\frac{Ea}{RT})$$

The estimated activation energies for all four different samples are given in the Table 1. 

It is evident from the conductivity measurements that the rapid dynamics of protons was facilitated by the supramolecular assembly and co-assembly of organic phosphonic acid **1** and the proton facilitators, where long range ionic channels act as a tunnel allowing the hopping of protons in a cooperative manner. In the literature, some materials with similar ionic channels have been reported with comparable proton efficiencies. However, on the contrary, the proton conduction in these materials seems to be taking place in three dimensions (S2) [33,34]. 

It should be noted that neither the organic phosphonic acid **1** nor any of the proton facilitators (pyrazole, triazole or benzimidazole) exhibit high proton conductivity when existing as separate entities. Only their self-assembly into molecular columns can endow them with the ability to exchange protons in a holistic and cooperative way, where the protons are assisting the self-assembly as well as raising the conductivity significantly via a hydrogen bonded network.

Further correlation between efficient proton conduction and the columnar morphology of these molecular wires was established by 2DWAXS experiments. X-ray results for the pure organic phosphonic acid **1** and its self-assembly with different proton facilitators, along with the illustration of bundles of the columns or molecular wire containing ionic pores are given in Figure 3A,B, respectively. The characteristic patterns of the columnar type of morphology measured from the organic phosphonic acid **1** at 30 °C point toward a greater extent of crystalline order in the molecular assembly (Figure 3a). The lattice could not be obtained in the crystalline phase, as the discs of molecules in columns were tilted by ca. 40° towards the columnar axis. Organization of the molecules into a columnar shape occurs by the disc-like form of molecules forming due to hydrogen bonds, followed by their arrangement via π-stacking interactions, then local phase separation occurring between the aromatic part and aliphatic constituents forming columns. For the organic phosphonic acid **1**, an intra-columnar period of 0.54 nm with a π-stacking distance of 0.41 nm was determined. Interestingly, as the phosphonic acid groups reside inside the center of columns, they lack any percolation pathway in two dimensions. The location of these phosphonic acid groups in the axis of the molecular columns suggests that the ionic pathway for proton conduction is exclusively 1D. Another point to note is that the interface between the phosphonic groups and surface of the electrode can be altered by the orientation of molecular columns and; hence, this could have a noticeable influence on the intrinsic proton conductivity. Furthermore, any defects in the columns of the molecules cannot be disregarded either when arguing about the proton conductivity.

Further morphological investigations of the molecular wires were carried out in order to study the effect of the organic proton facilitators on the molecular organization of organic phosphonic acid **1,** and to correlate with the proton conduction (Figure 3a–d) [35]. The diffraction patterns for compound **1** with pyrazole, triazole and benzimidazole are depicted in Figure 3a–d, respectively. For comparison, the diffraction patterns for the separate proton facilitators are also provided (Figure S2) [S1]. For the mixture of compound **1** with pyrazole, an increased intercolumnar period of about 3.62 nm and intercolumnar distance of 0.40 nm was observed, occurring due to the accommodation of pyrazole molecules in the axis of the columns (Figure 3b). However, the pi-pi distances were slightly reduced due to the formation of hydrogen bonds between the phosphonic acid and nitrogen of the pyrazole; hence, the arrangement of the molecules was slightly disturbed. By comparing the diffraction pattern of the free pyrazole molecules, as shown in SI, it is evident that the pyrazole molecules were accommodated within the ionic channels. In the 2D wide angle X-ray patterns of the mixture of compound **1** and the triazole (Figure 3c), the appearance of sharp scattering indicated the strong packing of the molecules via hydrogen bonds associated with the crystalline structure of triazole. The intercolumnar period also increased to 3.73 nm. A large intercolumnar period of about 4.51 nm was observed also for the mixture of compound **1** and benzimidazole, suggesting that the bigger size of the guest molecules (benzimidazole) can disturb the packing of the columns significantly. Nevertheless, the proton conductivity at 30–80 °C was found to increase for the mixture of organic phosphonic acid **1** and pyrazole, this could be related to the tendency of the proton facilitator such as pyrazole to fit in the axis of the columns due to its small size. On the other hand, the large proton facilitator, i.e., benzimidazole, has shown the propensity to disturb the organization of the molecules due to its chemical structure (Figure 3d and Figure S2d). Further information about the diffraction patterns of free benzimidazole molecules support the above theory very well (Figure S2).

**Figure 4:**
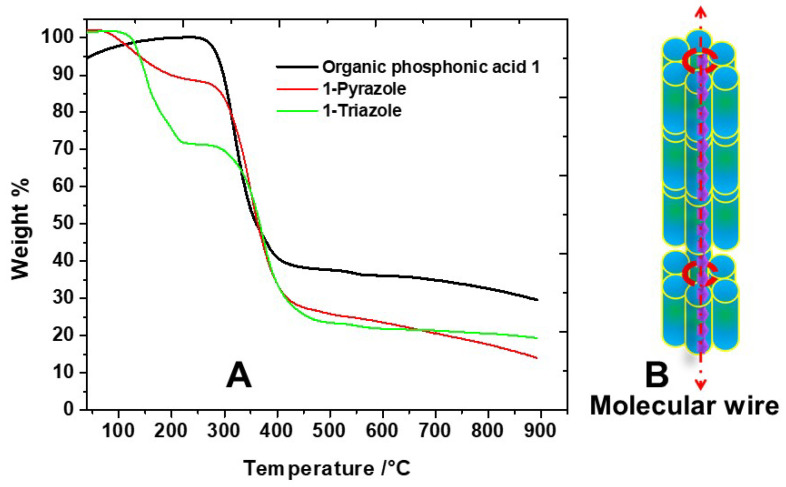
(**A**) Thermogravimetric analysis (TGA) (under N_2_) of molecular wires and (**B**) molecular wire with aromatic core and ionic channels.

Thermal stability is an essential requirement for the proton conductors working at high temperatures for battery applications [36,37,38,39,40,41,42]. To examine this, all molecular wires with different proton facilitators described here were investigated by thermal gravimetric analysis (TGA) to determine the weight loss in relation to change in temperature (Figure 4). The pure organic phosphonic acid **1** was found to be stable up to 250 °C (Figure 4), followed by a slight weight loss at 120 °C that was attributed to pyrazole and some residual solvent that may have been generated as a result of condensation between the phosphonic acid groups (Figure 4, red curve). A similar case was observed for the molecular wires of compound **1** with triazole (Figure 4, green curve). All acid–base composites were less stable when compared to the pure organophosphonic acid **1**. The weight loss occurring at 190 °C was associated with the decomposition of the organic proton facilitators. Noticeably, further decomposition at 330 °C appeared for the molecular wires of 1 and benzimidazole, which points towards free molecules (removed from ionic channels) of benzimidazole and thereby further supports our view that its assembly with organic phosphonic acid **1** is weak and its corresponding proton conductivity is the lowest (Figure S3) [S1]. 

## 3. Conclusions

In conclusion, thermally stable molecular wires are fabricated via self-assembly and co–assembly of two different small molecules, namely organic phosphonic acid and proton facilitators, producing a columnar morphology enabling rapid proton conduction in a dry state. In these molecular columns or wires, the proton conduction is increased as a result of long range ionic channels containing the molecules of proton facilitators that take part in a ‘cooperative’ proton exchange, even at high temperature (>100 °C). The proton conductivity of 10^−2^ S/cm for the molecular wires with pyrazole is attributed to the more feasible and faster proton dynamics in the hydrogen bonded network formed by the molecules. The proton conductivity followed the Arrhenius trend. The activation energies suggest the Grotthuss type of mechanism (proton hopping) for proton transportation occurring within the ionic channels in which no carrier moiety is involved. The formation of a columnar morphology containing ionic channels is evidenced by 2DWAXS and is associated with one-dimensional (1D) proton conduction taking place in these molecular wires. These molecular wires have demonstrated a precise correlation between the increased proton conduction and columnar morphology. These molecular wires offer further prospects for developing proton conducting membranes containing fabricated and stable interconnected ionic channels for energy applications. In the future, further focus will be on freezing these molecular wires in a free-standing film for achieving the proton conductivity of 10^−1^ S/cm in a dry state, as needed for commercial applications. In addition, a contrasting molecular system in which long chain alkylated and symmetrical proton facilitators will be used as phase separators to generate similar molecular columns and assemblies together with the organic phosphonic acid will be explored.

## 4. Experimental

### 4.1. Materials

All materials were purchased from Fluka (Buchs, Switzerland) and Sigma Aldrich (Saint Louis, MO, USA). 1, 2, 4-Triazole (Fluka, Buchs, Switzerland, 99%), pyrazole (Fluka, Buchs, Switzerland, 98%) and benzimidazole (Fluka, Buchs, Switzerland, 98%).

Synthesis of 3, 4, 5-tris(dodecyloxy) benzyl phosphonic acid (Organic phosphonic acid **1**) and the preparation of the blend with 1 and proton facilitator 

The synthesis of 3, 4, 5-tris(dodecyloxy) benzyl phosphonic acid **1** was carried out as described in the literature [15]. The complexes of compound **1** and the pyrazole, triazole and benzimidazole were prepared as follows. In general preparation, the compound **1** and azole were mixed in 1:2 *w*/*w* ratios and dissolved in a 1:1 ratio of dichloromethane and acetone in order to have absolute mixture of both. The solvents were removed and the resulting mixture was dried under vacuum. 

### 4.2. Film or Membrane Formation on the Substrate

The film or membrane of these molecular wires with columnar structures was deposited on the electrode surface directly by following the drop-casting process. In this process, a certain concentration of these supramolecular assemblies was taken into a suitable solvent such as dichloromethane. From the solution, the film was cast on the electrode surface by the drop-casting method. The film was dried for several hours at room temperature and then in a vacuum oven to remove any residual solvent. These films fabricated on the surface of the electrode were then utilized for the proton conducting analysis. 

### 4.3. Proton (Anhydrous) Conductivity Measurements

For the proton conductivity measurements, the samples for all complexes were prepared as follows. About 80 mg of the complex was placed above the electrode and was melted. Four glass fibers with the thickness of 0.1 mm were also placed along with the samples to avoid the contact with the electrodes. Afterwards, the second electrode was placed above this. The electrodes were connected with an impedance instrument. All proton conductivity measurements were done under dry N_2_. The thickness of the sample and the area of the electrodes were also measured for use in calculating the proton conductivity. The temperature dependence of conductivity was measured in the frequency range from 10^−1^ to 10^6^ Hz with the temperature ranging from 20 to 160 °C with an increment of 10 °C.

### 4.4. Thermogravimetric Analysis (TGA)

Thermal stability of all complexes was evaluated by thermogravimetric analysis (TGA) on a Perkin Elmer TGA 7 thermogravimetric analyzer (10 K/min heating rate in nitrogen atmosphere). All TGA trace data were obtained from 80 to 500 °C.

## Figures and Tables

**Table 1:** Activation energies and related proton conductivities of molecular wires with different proton facilitators.

	Molecular Wires	Anhydrous Proton Conductivity	Activation Energy (*Ea*)
1	Pure compound 1	8 × 10^−7^ S/cm	0.34 eV
2	Compound 1/Pyrazole	2 × 10^−3^ S/cm	0.12 eV
3	Compound 1/Triazole	1 × 10^−3^ S/cm	0.41 eV
4	Compound 1/Benzimidazole	5 × 10^−2^ S/cm	0.51 eV

## Data Availability

Not applicable.

## Supplementary Materials

The following supporting information can be downloaded at: https://www.mdpi.com/article/10.3390/membranes12121174/s1, supplementary data s1, FT-IR spectra, 2DWAXS patterns, TGA, Tables for properties.

## Abbreviations

2DWAXS	2-Dimensional Wide Angle Scatterings
TGA	Thermogravimetric analysis
FT-IR	Fourier-transform infrared
