# Supplementary material for: Proton Conducting Membranes with Molecular Self Assemblies and Ionic Channels for Efficient Proton Conduction
Source: Membranes (Basel). 2022 Nov 22;12(12):1174. doi: 10.3390/membranes12121174 (PMC9781519; doi:10.3390/membranes12121174)
Supplement: Supplementary file 1 [file membranes-12-01174-s001.zip › membranes-2004397-supplementary.pdf]

Table S1. Properties of organic heterocycles used as proton facilitators

Organic base	mp °C	bp °C	Acidity (*p*Ka)
Pyrazole	68	168-188	2.5
1H-1, 2, 4-Triazole	120	260	2.2
Benzimidazole	170	360	8.52

Table S2. Anhydrous proton conductivities for other molecular systems with ionic channels reported in the literature

S.N.	Anhydrous proton exchange materials[a]	Anhydrous proton conductivity[b]	Temperature
1.	Phosphonated self-assembled system[c]	10^{-2} S/cm	140 °C
2.	Self-organized molecular system[d]	10^{-3} S/cm	150 °C
3.	Polymers with the ionic channels[e]	2.4×10^{-6} S/cm	90 °C
4.	Imidazole-containing triblock polymers[f]	9.7×10^{-4} S/cm	240 °C

[a]Polymeric or nonpolymeric membranes reported in the literature; [b] Proton conductivity in the absence of water
[c, d] Reference no. 15 and 16 in main manuscript
[e]Tuning anhydrous proton conduction in single-ion polymers by crystalline ion channels, Onnuri Kim, Kyoungwook Kim, U. Hyeok Choi, Moon Jeong ParkNat Commun 9, 5029 (2018)
[f]Chainika Jangu, Jing-Han Helen Wang, Dong Wang, Gregory Fahs, James R. Heflin, Robert B. Moore, Ralph H. Colby, Timothy E. Long, Imidazole-containing triblock copolymers with a synergy of ether and imidazolium sites, J. Mater. Chem. C, 2015,**3**, 3891-3901

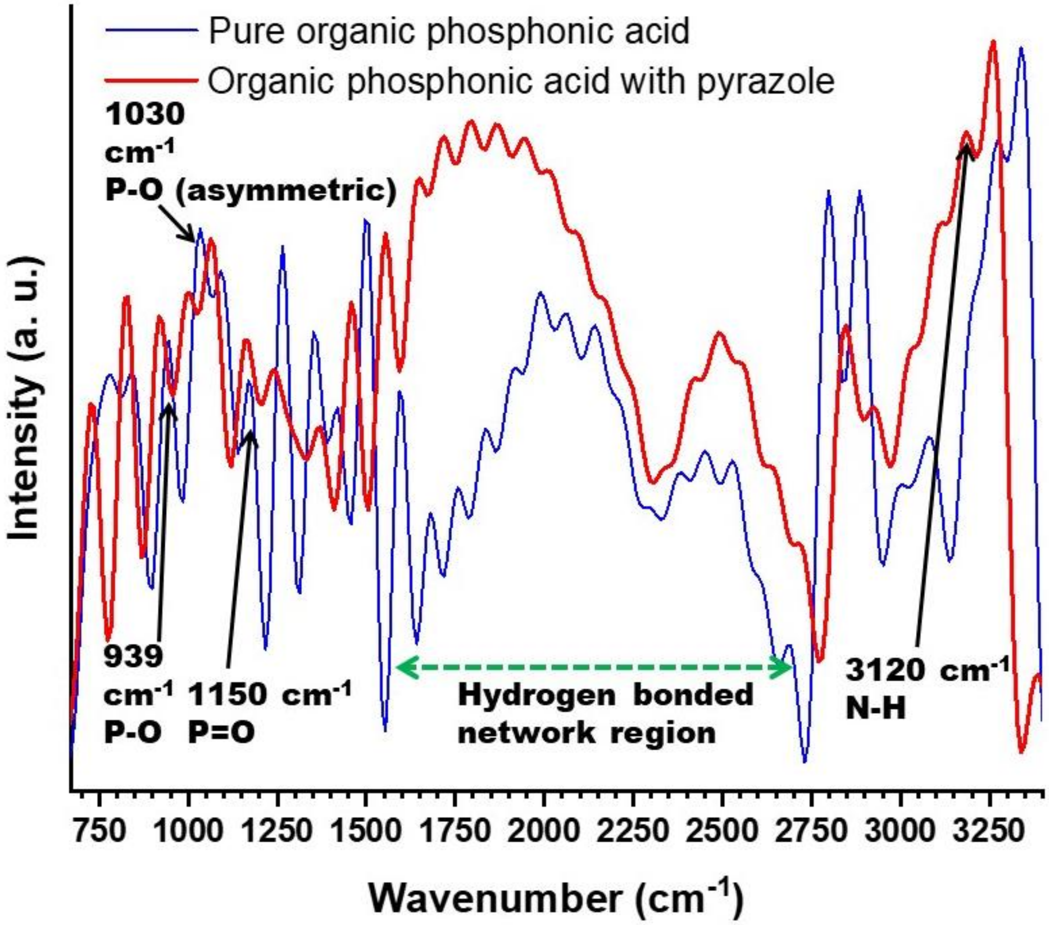

Figure S1 FT-IR spectra of (blue line, before complex formation) pure organophosphonic acid 1 and (red line, after complex formation) the acid-base composite of the organophosphonic acid 1 and pyrazole.

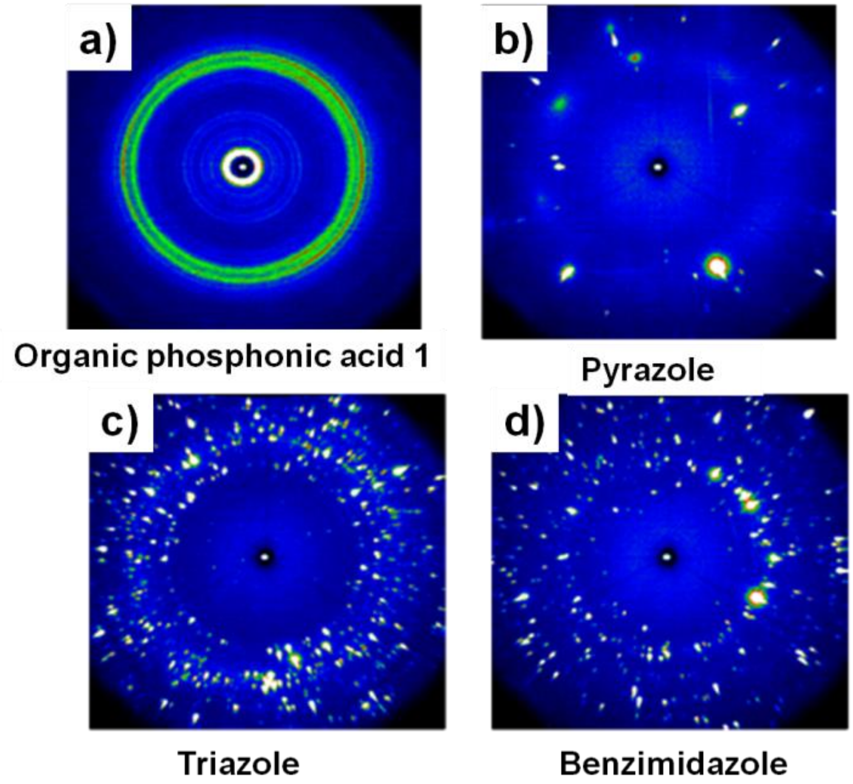

Figure S2 2DWAXS patterns of a) organic phosphonic acid 1, b) pyrazole, c) triazole, and d) benzimidazole before mixing them together.

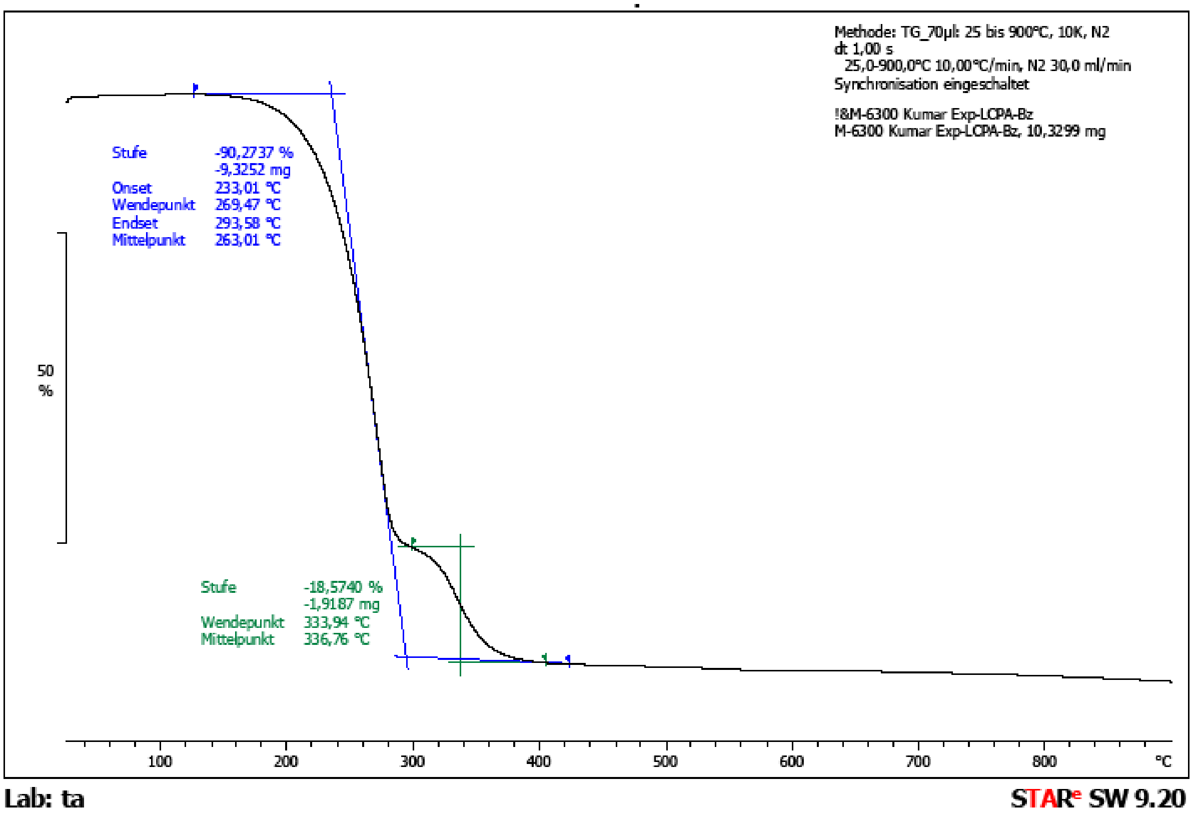

Figure S3 Thermogravimetric analysis of molecular wires with benzimidazole under N_2.
